# Obituary

**Published:** 1902-12-15

**Authors:** 


### OBITUARY.

### JOHN A. HARRIS.

John A. Harris was born at Preston, Conn., May 27th, 1833. When about twenty years of age he commenced the study of dentistry at Norwich, Conn., with Dr. Parkhurst, with whom he remained for several years. Finally believing that the west afforded better opportunities, he came to Michigan in the spring of 1855, and after spending some time as a student in Pontiac, and as a practitioner in East Saginaw and Bay City, he bought the dental practice of Dr. William Cahoon, at Pontiac, in the spring of 1857. From that time Pontiac was his home and he practiced there continuously until 1901. He was married June 3rd, 1837, to Jane H. Cooper, of Sterling, N. Y., who died October 3rd, 1893. He was survived by three children, Mrs. Thomas B. Bronson, Miss Pauline Harris and Williams C. Harris.

In his fifty years of practice Dr. Harris became widely known through Michigan, especially in Oakland County where he was acquainted with almost the entire community in the early days. He was at one time an alderman of the city of Pontiac, and was for many years prominently connected with the Methodist Church and the Masonic fraternity.

In 1900 his health began to fail and he was finally obliged to retire from practice in 1901, and thereafter lived with his son in Detroit. A long illness followed, and he died at Detroit, August 9th, 1902, the immediate cause being Addison's disease. He was buried with Masonic honors at Pontiac.

